# Remarkable Case of Injury from Glass

**Published:** 1873-06

**Authors:** George W. Kittell

**Affiliations:** Shabbona, Ill.


ARTICLE II.—*Remarkable Case of Injury from Glass.* By GEORGE W. KITTELL, M.D., Shabbona, Ill.

The following case, of which I am induced to give a brief account, is a real curiosity. Its rarity, and, almost tragic events, have been a daily source of wonder and excitement to our community. Space will not allow me to give the details of all that occurred to my patient during a period of eight years. It would fill a volume. This account will be deficient in many minor particulars, as no notes were kept of the case.

December 24th, 1864, a young lady named Caroline Low cut her hand severely while replacing a window. She intended striking the top of the sash with her fist, but, missing it, the hand passed through the upper pane, about three inches from its lower edge. The glass broke diagonally; three triangular pieces entering the ulnar border of the hand opposite the middle of the palm. The external wound was one and a fourth inches in length, the same as the base of the longest piece; so they must have slid one upon the other as they entered.

On inquiry, Miss Low told me the glass reached nearly across the palm. To mitigate the pain she pressed the right hand firmly between the thumb and fingers of the left, thereby breaking the glass in many pieces. She obstinately refused to have medical aid until three weeks had elapsed, when it was feared she would lose the hand.

Dr. Z. C. Brown was called. He found the hand and arm enormously swollen. He made several incisions in the palm between the fingers, and plugged the wounds with lint. The hand was so swollen that no large pieces could be detected. Topical applications to reduce the swelling were made. On the following day the lint was removed, and was found covered by shining particles of glass, many of them smaller than a pin's head. This operation was repeated several times with like results, and the attendant thought the glass was all out. She was then for a short time under the care of Dr. S. N. Fish, who pursued the same treatment with like results.

May 1st, 1865, she came under the notice of the writer. The actual amount of glass in the hand was not even suspected, and though on full inquiry I became convinced that the amount was large, I was not much less surprised than others at the result. If the broken pane had been carefully examined at the time the accident occurred, it would have conclusively settled a vexed question.

Subsequently when the pieces actually removed, from the crown of her head to the soles of her feet, were numbered by thousands, no wonder the credulity of the public was sorely tried. Some of the plebeians thought there was nothing miraculous in it. Some said she swallowed the glass; others that it was produced in the system by medicines I had given her; others still, that the doctor pounded up the glass at home, and reported that he took it from the patient for a sensation.

At the time I saw the patient first the wounds were all healed, leaving the hand contracted and distorted. I made an incision over a tender spot near the thumb, and filled the cut with lint. I counted twenty pieces on it next day when it was removed; they were larger than any before obtained, and were preserved.

The patient suffered considerable pain, and came to my office every two weeks to be lanced; much glass was removed in this way, the wounds healing with remarkable rapidity.

In August, this year, a large superficial sore appeared at the elbow, which was dressed with lint and simple cerate. A large number of small particles of glass were removed on the lint and with small forceps. The wound continued to discharge glass for four months, when it healed up. In the November following, a larger sore appeared at the shoulder with the same history.

During the spring of 1866, a large sore, in all respects like the others, made its appearance on the upper part of the chest.

In the following summer, glass was removed from the scalp, and numerous pieces worked their way into the eye through the conjunctiva, causing intense pain.

In January, 1867, the soles of the feet became sore, and some glass was discharged. The feet were lanced and the wounds plugged with cotton. She was soon severely attacked with tetanus, which continued two weeks. She was treated by large and continued doses of morphine. Sixty grains were given, in grain doses, in six days.

When she had been free from spasm two weeks, another attempt was made to remove glass from her feet, which was followed as before by tetanus. Large doses of morphine were again given, which controlled the spasms.

The attack continued seven weeks. One cause of her prolonged sickness was, two elderly female friends who would not administer the medicine in my absence, saying so much morphine would kill her. They could not see that it would be as well to die of morphine as tetanus. She received another shock from the visit of an honest clergyman, who in spite of my remonstrance repeated his visit.

Her convalescence, if it might be so termed, was tedious, and for a long time she was so sensitive that cutting the hair or nails would bring on a convulsion. She improved very slowly.

In September and October, 1867, she complained of pain in the head and neck; turning the head suddenly would bring on a convulsion. The first piece of glass large enough to show two polished surfaces, was at this time taken from the neck. Shortly after, a pointed piece half an inch long was removed from the ear by the forceps. The inflammation and suppuration which followed, destroyed the power of hearing in that ear.

Immediately after this, a piece of glass five-eighths of an inch long cut its way into the œsophagus and was removed by an emetic. Afterward, on each of two successive days, two pieces were removed in the same manner. One, the largest ever obtained, was one and one-fourth inches in width. The four pieces fitted exactly, the whole forming a triangle two and three-fourths inches in length. Her throat was badly cut in the removal of these large pieces, and bled profusely. When the wounds healed, the canal was so much contracted that the swallowing of solids was ever after difficult. From this time about twenty pieces of various sizes and shapes were removed.

Until September, 1868, nothing unusual occurred except the expectoration of blood with some small particles of glass. The glass probably cut into the pharynx above and thus found entrance into the lungs and were thrown off as any other foreign substance.

At the above-mentioned date she was stopping at a hotel in Shabbona. The room next hers was occupied by a dentist, who was one of a horse-stealing gang. She overheard them in their deliberations, of which they by some means became aware and undertook to destroy her by poisoning.

She was taking morphia at the time, the powders being kept on a shelf in her room. Some one entered the room in her absence, took out the morphine and placed arsenic in the same papers. I was sent for in haste, and found my patient with unequivocal symptoms of arsenical poisoning. Vomited her freely and administered magnesia as an antidote. She soon recovered. To prevent suspicion on the part of the rascals, I told the friends she had cholera morbus, and my statement was confirmed by a relative who is a physician and saw her next day.

Further attempts were made with strychnia as had been done with arsenic. The medicine was placed on the shelf as before, and regularly removed by the patient who gave them to me. The medicine she actually took was kept in another place. I tested the powders taken from the shelf and all contained strychnia.

As soon as she was able she prepared to return home. She placed her medicine in a small satchel, and laid it on a table. She left the room for a moment only, but events proved that some one was on the watch, for the first powder she took after going home produced such violent symptoms as to leave no doubt as to the cause. I happened to call in a short time, but unfortunately had no antidote with me, and I was obliged to send three miles for medicine. The inflammation which followed nearly destroyed her; her convalescence was slow, and she never fully recovered.

Although the glass had been nearly all removed, her naturally strong constitution was wrecked, and there was a condition of general irritability and nervous prostration. The irritation of occasional particles of glass, of over exertion, or exposure, would bring on fever, delirium and convulsions, so that she required almost constant medical attention. Her condition gradually improved until July, 1872.

It seems as if fate continually pursued her with some dire and unusual affliction; and had she not possessed an iron constitution, she must have succumbed long ere she did.

In July, 1872, while eating greens, she swallowed a potatoe bug and its eggs, which most unfortunately had escaped the cook's notice. She was not aware that anything was wrong until she was taken with fever, pain in the stomach, vomiting, etc. I was not sent for until next day. I learned that she had vomited frequently, and supposing that the offending matter had been removed, I administered remedies to check the vomiting, and subdue the inflammation. When I made my next visit I became satisfied as to the cause of the difficulty, and gave an emetic, followed by copious draughts of warm water, and on a careful examination of the matters ejected, was able to discern legs and other parts of the bug. The inflammation subsided. Her disordered nervous system was unable to sustain the shock, and spinal irritation was induced. She was treated with counter-irritants, tonics and anodynes. There was at times temporary improvement, but she grew worse in November, and died of exhaustion, December 4, 1872.

Thus ended a most unusual and remarkable case after almost eight years of intense suffering. No one could have suffered more. A small particle of dirt, all of us know, is a very unpleasant thing to have in the eye; but how trifling a matter it is compared to a piece of glass, with sharp cutting edges in every direction. I have merely given the outlines, as a more minute account would require too much space; but one more curious phase of her history should be noticed. Similar cases are on record, and this will serve somewhat to confirm them. I refer to clairvoyance.

In this case it was not produced by mesmerism, but by chloroform, and she became more and more susceptible to its influence. In the later stages of the case, this state came on occasionally from over excitement.

Before the accident which introduced the case, she was given chloroform for the purpose of having a tooth extracted. The doctor who administered it, had not always kept that moral rectitude, in some particulars, which becometh a physician. Shortly after the inhalation commenced she began to upbraid him for his conduct. The doctor was frightened, and accused a man, the only one beside himself who knew the circumstance, of telling. The man protested that he was innocent, for he really was. When Miss Low returned to consciousness she knew nothing of what she had said, or of the occurrence she had related.

My first knowledge of this effect of chloroform on her came in this way: After removing some glass one day, and while she was still under the influence of the anæsthetic, I was called out for a private interview. The weather being pleasant, we stepped into the orchard and sat down under a tree. When I returned she remarked, "you thought yourselves very cute when you went into the orchard to talk; but I heard it all." I then asked her to tell what she heard, and she related our conversation correctly. She had not left the bed in my absence, and could not see the orchard, as it was on the other side of the house, neither had she been told that I left the house. In fact, she was apparently unconscious the whole time, and when she had fully recovered from the influence of the chloroform, she knew nothing of what had been done or said. I had known her to say strange things while anæsthetized, but until now had not understood it.

Sometimes, after having taken chloroform, she would rise in her sleep and go miles, in her night clothes, to find articles that had been lost. She never had any knowledge of these nocturnal expeditions in her waking state, except the proof afforded by the presence of missing articles, and the condition of the bed in the morning.

Her clairvoyant state was another existence to her. When in this state she would tell anything that had transpired at other times, while in the same condition. I have given her chloroform in order to enable her to find lost articles, which she could always do. Some little thefts, and sometimes bigger ones, were made known in the same way.

When very sick she was often delirious, sometimes for hours, which led many people to suppose she was insane, and some said she was possessed of the devil. It was from this fact that the horse thieves escaped punishment; many would take oath in court against her sanity. She was the principal witness, and popular prejudice, backed by some physicians, for no laudable purpose, carried the day.

To relate all that she said and did, while clairvoyant, would make a long and interesting chapter. The most interesting occurrences of this kind must be omitted because of their length. If any doubt is entertained as to the truth of these statements, any further proof desired will be gladly furnished by the author.

